# Tris{2-[4-(2-pyrid­yl)pyrimidin-2-ylsulfan­yl]eth­yl}amine

**DOI:** 10.1107/S160053680902741X

**Published:** 2009-07-18

**Authors:** Jian-Quan Wang, Ya-Wen Zhang, Lin Cheng

**Affiliations:** a School of Chemistry and Chemical Engineering, Southeast University, Nanjing 211189, People’s Republic of China

## Abstract

The tripodal character of the title compound, C_33_H_30_N_10_S_3_, arises from the three thio­ether arms surrounding a central amine N atom. The three arms have approximately the same conformation but distinct geometries in a *trans–trans–cis* conformation, resulting in a short pyridine–sulfanyl N⋯S distance of 4.320 (7) Å. The distances of the central N atom to the N atoms of three pyridine rings in the arms are 8.305 (7), 8.032 (7) and 5.076 (9)Å. In the crystal, mol­ecules are joined into a three-dimensional supra­molecular network *via* effective π–π stacking between adjacent heterocycles [centroid–centroid distances of 3.700 (3)–4.118 (4) Å between adjacent inter­layer pyrimidine rings and 3.676 (4) Å between the pyridine rings].

## Related literature

For the use of tripodal ligands in crystal engineering, see: Hammes *et al.* (1998 ▶); Hiraoka *et al.* (2005 ▶). For the use of thio­ether ligands in crystal engineering, see: Dong *et al.* (2008*a* ▶,*b* ▶); Zhang *et al.* (2008 ▶).
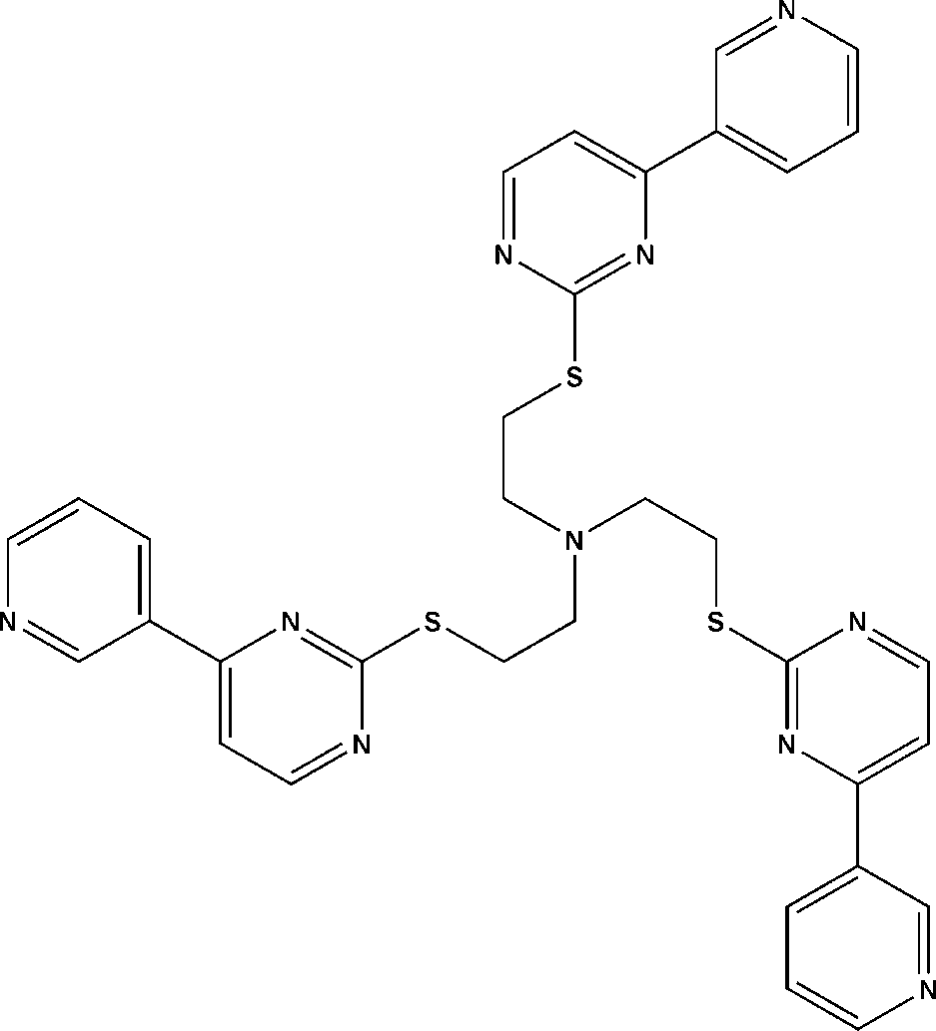


## Experimental

### 

#### Crystal data

C_33_H_30_N_10_S_3_
*M* *_r_* = 662.85
Orthorhombic, 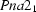

*a* = 16.169 (3) Å
*b* = 25.670 (5) Å
*c* = 7.6166 (14) Å
*V* = 3161.3 (10) Å^3^
*Z* = 4
Mo *K*α radiation
μ = 0.28 mm^−1^
*T* = 295 K
0.30 × 0.20 × 0.20 mm

#### Data collection

Bruker SMART CCD diffractometer
Absorption correction: multi-scan (*SADABS*; Sheldrick, 2000 ▶) *T*_min_ = 0.922, *T*_max_ = 0.947
16502 measured reflections
6104 independent reflections
3099 reflections with *I* > 2σ(*I*)
*R*_int_ = 0.105

#### Refinement

*R*[*F* ^2^ > 2σ(*F* ^2^)] = 0.070
*wR*(*F* ^2^) = 0.123
*S* = 1.06
6104 reflections
415 parameters
1 restraint
H-atom parameters constrained
Δρ_max_ = 0.41 e Å^−3^
Δρ_min_ = −0.25 e Å^−3^
Absolute structure: Flack (1983 ▶), 2750 Friedel pairs
Flack parameter: −0.33 (12)

### 

Data collection: *SMART* (Bruker, 2000 ▶); cell refinement: *SAINT* (Bruker, 2000 ▶); data reduction: *SAINT*; program(s) used to solve structure: *SHELXS97* (Sheldrick, 2008 ▶); program(s) used to refine structure: *SHELXL97* (Sheldrick, 2008 ▶); molecular graphics: *SHELXTL* (Sheldrick, 2008 ▶); software used to prepare material for publication: *SHELXL97* and *PLATON* (Spek, 2009 ▶).

## Supplementary Material

Crystal structure: contains datablocks I, global. DOI: 10.1107/S160053680902741X/si2186sup1.cif

Structure factors: contains datablocks I. DOI: 10.1107/S160053680902741X/si2186Isup2.hkl

Additional supplementary materials:  crystallographic information; 3D view; checkCIF report

## Figures and Tables

**Fig. 1.**
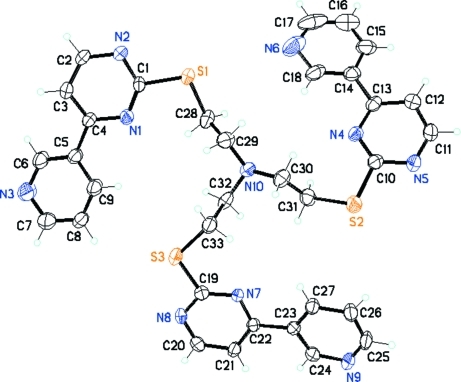
The molecular structure of the title compound with labelled non-hydrogen atoms. Displacement ellipsoids are drawn at the 20% probability level.

**Fig. 2.**
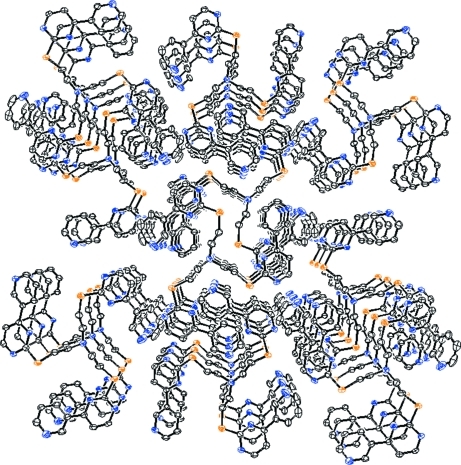
A section of the three-dimensional supramolecular network of the title compound viewed down the c-axis.

## supplementary crystallographic information

### Comment

Recently, tripodal ligands have attracted much attention because they can be used to construct interesting topologies such as moleular cages and boxes (Hammes *et al.* 1998, Hiraoka *et al.* 2005). Meanwhile, flexible thioethers have been well established ligands in coordination and metallosupramolecular chemistry because of their rich structural information (Dong *et al.* 2008*a, b*; Zhang *et al.* 2008). Herein, we report the crystal structure of the title compound, C_33_H_30_N_10_S_3_, based on a tripodal dithioether ligand -tris(2-(4-(pyridin-3-yl)pyrimidin-2-ylthio)ethyl)amine.

The tripodal character of the title compound arises from the three thioether arms surrounding a central amine nitrogen. The three arms have approximately the same conformation but distinct geometries, in a *trans-trans-cis* conformation (Fig. 1). The distances of the central nitrogen atom to the nitrogen atoms of three pyridine rings in the arms are 8.305 (7), 8.032 (7) and 5.076 (9)°, respectively. Two different heterocyclic rings in each arm are not coplanar with the angle of 18.0 (3), 11.9 (3) and 26.4 (2)°, respectively. The discrete molecules are joined into a three-dimensional supramolecular network *via* effective π—π stacking (Fig. 2) between the interlayer adjacent pyrimidine rings with the Cg3···Cg5^i^ separation of 3.700 (3) to 4.118 (4) Å, with a torsion angle of 7.24°, and a Cg4···Cg6^ii^ distance between pyridine rings of 3.676 (4) Å, with a torsion angle of 4.64°. Cg3 and Cg5 are the centroids of the pyrimidine rings (N5 C11 C12 C13 N4 C10) and (N8 C20 C21 C22 N7 C19), and Cg4 and Cg6 are the centroids of the pyridine rings (C14 C15 C16 C17 N6 C18) and (C23 C24 N9 C25 C26 C27). Symmetry codes: (i = 1 - *x*, 1 - *y*, 1/2 + *z*; ii = 1 - *x*, 1 - *y*, -1/2 + *z*).

### Experimental

An ethanol solution (50 ml) of tris(2-bromoethyl)amine (3.35 g, 10 mmol) was added to a dry ethanol solution (300 ml) containing 4-(pyridin-3-yl)pyrimidine-2-thiol (5.67 g, 30 mmol) and sodium hydroxide (1.20 g, 30 mmol). The solution was stirred and refluxed for 8 h. Yellow precipitates were filtered out, washed by water and ethanol, and dried in vacuum. Yield (3.24 g) 48.9%. The yellow crystals were obtained after the filter slowly evaporated.

### Refinement

All the H atoms were positioned geometrically and refined using a riding model with C—H = 0.93–0.97 Å and with *U*_iso_(H) = 1.2*U*_iso_(C).

### Crystal data

**Table:**

C_33_H_30_N_10_S_3_	*F*(000) = 1384
*M**_r_* = 662.85	*D*_x_ = 1.393 Mg m^−^^3^
Orthorhombic, *P**n**a*2_1_	Mo *K*α radiation, λ = 0.71073 Å
Hall symbol: P 2c -2n	Cell parameters from 787 reflections
*a* = 16.169 (3) Å	θ = 2.4–28.0°
*b* = 25.670 (5) Å	µ = 0.28 mm^−^^1^
*c* = 7.6166 (14) Å	*T* = 295 K
*V* = 3161.3 (10) Å^3^	Block, yellow
*Z* = 4	0.30 × 0.20 × 0.20 mm

### Data collection

**Table:**

Bruker SMART CCD diffractometer	6104 independent reflections
Radiation source: fine-focus sealed tube	3099 reflections with *I* > 2σ(*I*)
graphite	*R*_int_ = 0.105
φ and ω scans	θ_max_ = 26.0°, θ_min_ = 2.0°
Absorption correction: multi-scan (*SADABS*; Sheldrick, 2000)	*h* = −19→18
*T*_min_ = 0.922, *T*_max_ = 0.947	*k* = −25→31
16502 measured reflections	*l* = −9→9

### Refinement

**Table:**

Refinement on *F*^2^	Secondary atom site location: difference Fourier map
Least-squares matrix: full	Hydrogen site location: inferred from neighbouring sites
*R*[*F*^2^ > 2σ(*F*^2^)] = 0.070	H-atom parameters constrained
*wR*(*F*^2^) = 0.123	*w* = 1/[σ^2^(*F*_o_^2^) + (0.0045*P*)^2^ + 0.8*P*] where *P* = (*F*_o_^2^ + 2*F*_c_^2^)/3
*S* = 1.06	(Δ/σ)_max_ < 0.001
6104 reflections	Δρ_max_ = 0.41 e Å^−^^3^
415 parameters	Δρ_min_ = −0.25 e Å^−^^3^
1 restraint	Absolute structure: Flack (1983), 2750 Friedel pairs
Primary atom site location: structure-invariant direct methods	Flack parameter: −0.33 (12)

### Special details

**Table:**

Experimental. A trial to refine the structure by interchanging the C16 and N6 atoms resulted in slightly worse R-values and larger anisotropic displacement parameters. A further trial to treat the N6 pyridine as a rotational disordered group, using EADP and PART instructions did not show any improvement. The large negative Flack parameter may result from an unresolved twinning problem or from tiny intergrown material that contribute to additional intensities in the data set. This may also explain the relatively large Rint and R(sigma) values after absorption correction and data reduction.
Geometry. All e.s.d.'s (except the e.s.d. in the dihedral angle between two l.s. planes) are estimated using the full covariance matrix. The cell e.s.d.'s are taken into account individually in the estimation of e.s.d.'s in distances, angles and torsion angles; correlations between e.s.d.'s in cell parameters are only used when they are defined by crystal symmetry. An approximate (isotropic) treatment of cell e.s.d.'s is used for estimating e.s.d.'s involving l.s. planes.
Refinement. Refinement of *F*^2^ against ALL reflections. The weighted *R*-factor *wR* and goodness of fit *S* are based on *F*^2^, conventional *R*-factors *R* are based on *F*, with *F* set to zero for negative *F*^2^. The threshold expression of *F*^2^ > σ(*F*^2^) is used only for calculating *R*-factors(gt) *etc*. and is not relevant to the choice of reflections for refinement. *R*-factors based on *F*^2^ are statistically about twice as large as those based on *F*, and *R*- factors based on ALL data will be even larger.

### Fractional atomic coordinates and isotropic or equivalent isotropic displacement parameters (Å^2^)

**Table:**

	*x*	*y*	*z*	*U*_iso_*/*U*_eq_	
S1	0.83661 (8)	0.33092 (6)	0.0846 (3)	0.0824 (6)	
S2	0.41967 (9)	0.46398 (6)	0.0662 (3)	0.0677 (5)	
S3	0.78712 (8)	0.58482 (6)	0.0894 (3)	0.0695 (5)	
C1	0.9379 (3)	0.3547 (2)	0.1233 (9)	0.0600 (19)	
C2	1.0620 (3)	0.3334 (3)	0.2283 (10)	0.071 (2)	
H2	1.0985	0.3099	0.2795	0.085*	
C3	1.0900 (4)	0.3824 (2)	0.1879 (9)	0.0692 (19)	
H3	1.1450	0.3915	0.2062	0.083*	
C4	1.0356 (3)	0.4172 (2)	0.1206 (7)	0.0461 (15)	
C5	1.0572 (3)	0.4721 (2)	0.0830 (10)	0.0564 (15)	
C6	1.1378 (3)	0.4874 (3)	0.0554 (11)	0.077 (2)	
H6	1.1787	0.4619	0.0539	0.092*	
C7	1.1010 (4)	0.5729 (3)	0.0369 (11)	0.093 (3)	
H7	1.1157	0.6078	0.0236	0.112*	
C8	1.0201 (4)	0.5610 (2)	0.0618 (11)	0.080 (2)	
H8	0.9801	0.5870	0.0600	0.096*	
C9	0.9983 (3)	0.5105 (2)	0.0894 (10)	0.0723 (18)	
H9	0.9435	0.5020	0.1126	0.087*	
C10	0.3913 (4)	0.4001 (2)	0.0151 (8)	0.0567 (18)	
C11	0.2849 (4)	0.3452 (3)	−0.0115 (9)	0.074 (2)	
H11	0.2288	0.3377	−0.0033	0.089*	
C12	0.3360 (4)	0.3062 (3)	−0.0749 (9)	0.0715 (19)	
H12	0.3159	0.2739	−0.1098	0.086*	
C13	0.4198 (4)	0.3189 (2)	−0.0827 (7)	0.0521 (16)	
C14	0.4821 (4)	0.2805 (3)	−0.1415 (9)	0.0622 (18)	
C15	0.4630 (5)	0.2367 (3)	−0.2312 (10)	0.084 (2)	
H15	0.4077	0.2298	−0.2550	0.101*	
C16	0.5189 (7)	0.2033 (4)	−0.2863 (13)	0.105 (3)	
H16	0.5044	0.1741	−0.3516	0.126*	
C17	0.5973 (8)	0.2132 (4)	−0.2445 (14)	0.130 (4)	
H17	0.6364	0.1889	−0.2805	0.155*	
C18	0.5641 (5)	0.2892 (3)	−0.1054 (10)	0.083 (2)	
H18	0.5785	0.3194	−0.0452	0.099*	
C19	0.7194 (3)	0.6319 (2)	0.0044 (7)	0.0524 (16)	
C20	0.7130 (4)	0.7117 (2)	−0.1116 (8)	0.0663 (18)	
H20	0.7384	0.7419	−0.1517	0.080*	
C21	0.6267 (3)	0.7084 (2)	−0.1158 (8)	0.0607 (17)	
H21	0.5946	0.7356	−0.1588	0.073*	
C22	0.5919 (3)	0.6642 (2)	−0.0549 (7)	0.0412 (13)	
C23	0.5002 (3)	0.6563 (2)	−0.0429 (7)	0.0412 (13)	
C24	0.4472 (4)	0.6917 (2)	−0.1183 (9)	0.0606 (17)	
H24	0.4703	0.7185	−0.1842	0.073*	
C25	0.3355 (3)	0.6511 (3)	−0.0095 (9)	0.069 (2)	
H25	0.2784	0.6494	0.0051	0.082*	
C26	0.3812 (3)	0.6136 (2)	0.0675 (9)	0.0636 (17)	
H26	0.3566	0.5866	0.1302	0.076*	
C27	0.4666 (3)	0.6166 (2)	0.0496 (8)	0.0523 (16)	
H27	0.5004	0.5915	0.1008	0.063*	
C28	0.7832 (3)	0.3846 (2)	−0.0206 (9)	0.0719 (19)	
H28A	0.8232	0.4102	−0.0603	0.086*	
H28B	0.7532	0.3719	−0.1222	0.086*	
C29	0.7245 (3)	0.4093 (2)	0.1049 (9)	0.0671 (17)	
H29A	0.6929	0.3824	0.1639	0.080*	
H29B	0.7554	0.4283	0.1932	0.080*	
C30	0.5859 (3)	0.4472 (2)	0.1069 (8)	0.0635 (17)	
H30A	0.5698	0.4127	0.1462	0.076*	
H30B	0.5893	0.4699	0.2085	0.076*	
C31	0.5245 (3)	0.4676 (2)	−0.0191 (8)	0.0654 (19)	
H31A	0.5278	0.4478	−0.1273	0.078*	
H31B	0.5376	0.5036	−0.0461	0.078*	
C32	0.7063 (3)	0.4951 (2)	−0.0204 (7)	0.0576 (17)	
H32A	0.6712	0.5140	−0.1019	0.069*	
H32B	0.7587	0.4889	−0.0787	0.069*	
C33	0.7220 (3)	0.5299 (2)	0.1391 (8)	0.066 (2)	
H33A	0.7479	0.5094	0.2309	0.079*	
H33B	0.6695	0.5425	0.1837	0.079*	
N1	0.9562 (2)	0.40316 (17)	0.0839 (7)	0.0566 (12)	
N2	0.9844 (3)	0.31788 (18)	0.1973 (7)	0.0644 (15)	
N3	1.1603 (3)	0.5370 (2)	0.0307 (9)	0.097 (2)	
N4	0.4466 (3)	0.36629 (19)	−0.0387 (6)	0.0526 (13)	
N5	0.3090 (3)	0.39190 (19)	0.0383 (7)	0.0636 (15)	
N6	0.6259 (4)	0.2555 (3)	−0.1540 (10)	0.121 (3)	
N7	0.6377 (2)	0.62430 (16)	0.0073 (6)	0.0452 (12)	
N8	0.7597 (3)	0.6734 (2)	−0.0528 (7)	0.0618 (14)	
N9	0.3649 (3)	0.6902 (2)	−0.1038 (8)	0.0729 (16)	
N10	0.6680 (3)	0.44486 (18)	0.0158 (6)	0.0588 (14)	

### Atomic displacement parameters (Å^2^)

**Table:**

	*U*^11^	*U*^22^	*U*^33^	*U*^12^	*U*^13^	*U*^23^
S1	0.0393 (8)	0.0613 (10)	0.1466 (17)	0.0050 (7)	−0.0129 (14)	0.0014 (14)
S2	0.0528 (9)	0.0678 (11)	0.0827 (12)	−0.0013 (7)	0.0077 (11)	−0.0170 (12)
S3	0.0376 (7)	0.0688 (10)	0.1021 (13)	0.0047 (7)	−0.0071 (11)	0.0067 (13)
C1	0.033 (3)	0.055 (4)	0.092 (6)	−0.002 (3)	−0.011 (3)	−0.011 (4)
C2	0.044 (4)	0.057 (4)	0.112 (6)	0.012 (3)	−0.017 (4)	0.015 (4)
C3	0.041 (4)	0.067 (5)	0.100 (6)	0.000 (3)	−0.017 (4)	0.009 (4)
C4	0.039 (3)	0.051 (4)	0.048 (4)	0.008 (3)	0.000 (3)	0.005 (3)
C5	0.046 (3)	0.057 (4)	0.066 (4)	0.007 (3)	0.002 (4)	0.005 (4)
C6	0.048 (4)	0.074 (4)	0.109 (6)	0.004 (3)	0.010 (5)	0.027 (5)
C7	0.082 (5)	0.064 (5)	0.134 (8)	−0.002 (4)	−0.012 (5)	0.028 (5)
C8	0.049 (4)	0.063 (4)	0.129 (7)	0.011 (3)	−0.005 (5)	0.019 (5)
C9	0.047 (3)	0.067 (4)	0.103 (5)	0.001 (3)	0.002 (4)	0.027 (5)
C10	0.054 (4)	0.051 (4)	0.065 (5)	0.006 (3)	−0.002 (3)	−0.004 (3)
C11	0.045 (4)	0.070 (5)	0.106 (6)	−0.005 (4)	−0.009 (4)	0.012 (4)
C12	0.065 (5)	0.055 (4)	0.094 (6)	−0.001 (4)	0.004 (4)	0.007 (4)
C13	0.052 (4)	0.060 (5)	0.044 (4)	0.013 (3)	−0.001 (3)	0.014 (3)
C14	0.069 (5)	0.055 (5)	0.063 (5)	0.007 (4)	0.003 (4)	0.014 (4)
C15	0.100 (6)	0.053 (5)	0.100 (7)	0.008 (5)	0.013 (5)	−0.002 (5)
C16	0.147 (9)	0.075 (7)	0.094 (7)	0.015 (7)	0.007 (8)	0.004 (5)
C17	0.196 (12)	0.086 (8)	0.107 (9)	0.075 (9)	0.048 (9)	0.005 (6)
C18	0.082 (5)	0.068 (5)	0.098 (6)	0.034 (4)	0.021 (5)	0.008 (4)
C19	0.048 (4)	0.050 (4)	0.059 (5)	0.001 (3)	0.000 (3)	−0.007 (3)
C20	0.055 (4)	0.046 (4)	0.098 (5)	−0.014 (3)	0.009 (4)	0.006 (4)
C21	0.037 (3)	0.054 (4)	0.090 (5)	0.004 (3)	0.006 (3)	0.012 (4)
C22	0.040 (3)	0.043 (4)	0.041 (4)	−0.001 (3)	0.005 (3)	0.001 (3)
C23	0.041 (3)	0.044 (4)	0.039 (4)	0.002 (3)	0.002 (3)	0.001 (3)
C24	0.053 (4)	0.042 (4)	0.087 (5)	−0.006 (3)	−0.001 (4)	0.010 (4)
C25	0.031 (3)	0.087 (5)	0.088 (6)	0.000 (4)	0.001 (3)	−0.008 (4)
C26	0.044 (3)	0.071 (4)	0.076 (5)	−0.010 (3)	0.001 (4)	−0.005 (5)
C27	0.042 (3)	0.058 (4)	0.057 (4)	0.000 (3)	−0.003 (3)	0.005 (4)
C28	0.045 (3)	0.082 (5)	0.088 (5)	0.009 (3)	−0.012 (4)	0.001 (4)
C29	0.070 (4)	0.059 (4)	0.072 (5)	0.008 (3)	0.004 (4)	0.004 (4)
C30	0.059 (4)	0.073 (4)	0.058 (4)	0.012 (3)	−0.004 (4)	0.008 (4)
C31	0.061 (4)	0.066 (4)	0.069 (5)	−0.005 (3)	0.013 (4)	−0.002 (4)
C32	0.045 (4)	0.081 (5)	0.047 (4)	0.020 (3)	−0.005 (3)	−0.003 (4)
C33	0.044 (4)	0.068 (4)	0.085 (6)	0.004 (3)	0.001 (3)	0.020 (4)
N1	0.039 (2)	0.053 (3)	0.078 (4)	0.003 (2)	0.000 (3)	−0.018 (3)
N2	0.043 (3)	0.049 (3)	0.101 (4)	0.007 (3)	−0.010 (3)	0.007 (3)
N3	0.062 (4)	0.081 (4)	0.148 (7)	−0.002 (3)	0.017 (4)	0.030 (5)
N4	0.048 (3)	0.054 (3)	0.056 (3)	0.006 (3)	0.004 (3)	0.000 (3)
N5	0.044 (3)	0.065 (3)	0.082 (5)	0.003 (2)	0.001 (3)	0.006 (3)
N6	0.103 (5)	0.120 (6)	0.139 (7)	0.053 (5)	0.029 (5)	0.007 (5)
N7	0.031 (2)	0.040 (3)	0.064 (4)	0.001 (2)	−0.003 (2)	0.001 (2)
N8	0.033 (3)	0.056 (3)	0.097 (4)	−0.008 (3)	0.003 (3)	−0.004 (3)
N9	0.039 (3)	0.071 (4)	0.109 (5)	0.009 (3)	−0.009 (3)	0.002 (4)
N10	0.051 (3)	0.052 (3)	0.073 (4)	0.009 (3)	0.002 (3)	0.003 (3)

### Geometric parameters (Å, °)

**Table:**

S1—C1	1.773 (5)	C17—H17	0.9300
S1—C28	1.812 (6)	C18—N6	1.372 (8)
S2—C10	1.747 (6)	C18—H18	0.9300
S2—C31	1.818 (6)	C19—N8	1.324 (6)
S3—C19	1.754 (6)	C19—N7	1.334 (6)
S3—C33	1.799 (5)	C20—N8	1.318 (6)
C1—N1	1.312 (6)	C20—C21	1.399 (7)
C1—N2	1.333 (7)	C20—H20	0.9300
C2—N2	1.336 (6)	C21—C22	1.348 (7)
C2—C3	1.372 (8)	C21—H21	0.9300
C2—H2	0.9300	C22—N7	1.350 (6)
C3—C4	1.356 (7)	C22—C23	1.500 (7)
C3—H3	0.9300	C23—C27	1.353 (7)
C4—N1	1.361 (6)	C23—C24	1.373 (7)
C4—C5	1.479 (7)	C24—N9	1.337 (7)
C5—C9	1.371 (7)	C24—H24	0.9300
C5—C6	1.378 (7)	C25—N9	1.323 (7)
C6—N3	1.338 (7)	C25—C26	1.350 (7)
C6—H6	0.9300	C25—H25	0.9300
C7—N3	1.331 (7)	C26—C27	1.389 (6)
C7—C8	1.358 (8)	C26—H26	0.9300
C7—H7	0.9300	C27—H27	0.9300
C8—C9	1.359 (7)	C28—C29	1.489 (7)
C8—H8	0.9300	C28—H28A	0.9700
C9—H9	0.9300	C28—H28B	0.9700
C10—N4	1.312 (6)	C29—N10	1.459 (7)
C10—N5	1.358 (6)	C29—H29A	0.9700
C11—N5	1.317 (7)	C29—H29B	0.9700
C11—C12	1.385 (8)	C30—C31	1.477 (7)
C11—H11	0.9300	C30—N10	1.499 (6)
C12—C13	1.394 (7)	C30—H30A	0.9700
C12—H12	0.9300	C30—H30B	0.9700
C13—N4	1.335 (7)	C31—H31A	0.9700
C13—C14	1.479 (8)	C31—H31B	0.9700
C14—C15	1.352 (8)	C32—N10	1.457 (7)
C14—C18	1.372 (8)	C32—C33	1.530 (7)
C15—C16	1.314 (10)	C32—H32A	0.9700
C15—H15	0.9300	C32—H32B	0.9700
C16—C17	1.331 (12)	C33—H33A	0.9700
C16—H16	0.9300	C33—H33B	0.9700
C17—N6	1.367 (12)		
			
C1—S1—C28	104.5 (3)	C21—C22—N7	122.1 (5)
C10—S2—C31	102.3 (3)	C21—C22—C23	123.2 (5)
C19—S3—C33	104.6 (3)	N7—C22—C23	114.7 (5)
N1—C1—N2	129.9 (5)	C27—C23—C24	117.8 (5)
N1—C1—S1	119.8 (4)	C27—C23—C22	122.0 (5)
N2—C1—S1	110.3 (5)	C24—C23—C22	120.1 (5)
N2—C2—C3	122.9 (6)	N9—C24—C23	124.6 (6)
N2—C2—H2	118.5	N9—C24—H24	117.7
C3—C2—H2	118.5	C23—C24—H24	117.7
C4—C3—C2	118.4 (5)	N9—C25—C26	125.5 (6)
C4—C3—H3	120.8	N9—C25—H25	117.3
C2—C3—H3	120.8	C26—C25—H25	117.3
C3—C4—N1	120.9 (5)	C25—C26—C27	117.5 (6)
C3—C4—C5	123.3 (5)	C25—C26—H26	121.2
N1—C4—C5	115.8 (5)	C27—C26—H26	121.2
C9—C5—C6	117.3 (5)	C23—C27—C26	119.5 (5)
C9—C5—C4	120.9 (5)	C23—C27—H27	120.3
C6—C5—C4	121.6 (5)	C26—C27—H27	120.3
N3—C6—C5	123.3 (5)	C29—C28—S1	110.1 (5)
N3—C6—H6	118.3	C29—C28—H28A	109.6
C5—C6—H6	118.3	S1—C28—H28A	109.6
N3—C7—C8	122.9 (6)	C29—C28—H28B	109.6
N3—C7—H7	118.6	S1—C28—H28B	109.6
C8—C7—H7	118.6	H28A—C28—H28B	108.2
C7—C8—C9	119.1 (6)	N10—C29—C28	111.6 (6)
C7—C8—H8	120.4	N10—C29—H29A	109.3
C9—C8—H8	120.4	C28—C29—H29A	109.3
C8—C9—C5	120.0 (5)	N10—C29—H29B	109.3
C8—C9—H9	120.0	C28—C29—H29B	109.3
C5—C9—H9	120.0	H29A—C29—H29B	108.0
N4—C10—N5	127.4 (5)	C31—C30—N10	108.0 (5)
N4—C10—S2	120.7 (4)	C31—C30—H30A	110.1
N5—C10—S2	111.9 (4)	N10—C30—H30A	110.1
N5—C11—C12	125.6 (6)	C31—C30—H30B	110.1
N5—C11—H11	117.2	N10—C30—H30B	110.1
C12—C11—H11	117.2	H30A—C30—H30B	108.4
C11—C12—C13	115.2 (6)	C30—C31—S2	112.1 (4)
C11—C12—H12	122.4	C30—C31—H31A	109.2
C13—C12—H12	122.4	S2—C31—H31A	109.2
N4—C13—C12	121.2 (5)	C30—C31—H31B	109.2
N4—C13—C14	117.4 (5)	S2—C31—H31B	109.2
C12—C13—C14	121.3 (6)	H31A—C31—H31B	107.9
C15—C14—C18	117.2 (7)	N10—C32—C33	116.0 (4)
C15—C14—C13	123.4 (7)	N10—C32—H32A	108.3
C18—C14—C13	119.4 (6)	C33—C32—H32A	108.3
C16—C15—C14	123.1 (8)	N10—C32—H32B	108.3
C16—C15—H15	118.4	C33—C32—H32B	108.3
C14—C15—H15	118.4	H32A—C32—H32B	107.4
C15—C16—C17	117.0 (10)	C32—C33—S3	112.8 (4)
C15—C16—H16	121.5	C32—C33—H33A	109.0
C17—C16—H16	121.5	S3—C33—H33A	109.0
C16—C17—N6	126.5 (10)	C32—C33—H33B	109.0
C16—C17—H17	116.8	S3—C33—H33B	109.0
N6—C17—H17	116.8	H33A—C33—H33B	107.8
C14—C18—N6	123.1 (8)	C1—N1—C4	114.6 (5)
C14—C18—H18	118.4	C1—N2—C2	113.1 (5)
N6—C18—H18	118.4	C7—N3—C6	117.3 (5)
N8—C19—N7	127.6 (5)	C10—N4—C13	117.3 (5)
N8—C19—S3	111.6 (4)	C11—N5—C10	113.1 (5)
N7—C19—S3	120.8 (4)	C17—N6—C18	113.0 (8)
N8—C20—C21	122.4 (5)	C19—N7—C22	115.2 (5)
N8—C20—H20	118.8	C20—N8—C19	115.5 (5)
C21—C20—H20	118.8	C25—N9—C24	115.1 (6)
C22—C21—C20	117.2 (5)	C32—N10—C29	112.0 (4)
C22—C21—H21	121.4	C32—N10—C30	115.4 (4)
C20—C21—H21	121.4	C29—N10—C30	111.4 (5)
